# In vitro evaluation of Sensi-IP®: A soluble and mineralizing sensitivity solution

**DOI:** 10.1016/j.heliyon.2021.e08672

**Published:** 2021-12-25

**Authors:** Kathleen MacDonald, Effie Boudreau, Gavin Vaughan Thomas, Thomas Charles Badrock, Luke John Davies, Michael James Lloyd, Paul Steven Spradbery, Stephanie Turner-Cahill, Daniel Boyd

**Affiliations:** a IR Scientific Inc., Box 51, 1344 Summer St, Halifax, N.S, B3H 0A8, Canada; b Intertek Testing Services Ltd., Hooton, Cheshire, CH66 7NZ, UK; c Department of Applied Oral Sciences, Faculty of Dentistry, Dalhousie University, B3H 4R2, Canada

**Keywords:** Bioactive glass, Dentin hypersensitivity, Dentin occlusion

## Abstract

**Objectives:**

Sensi-IP®OG (SIP-OG) and Sensi-IP®FF (SIP-FF) are soluble bioactive glasses developed to treat dentin hypersensitivity and promote remineralization. Evaluation of their therapeutic potential to reduce dentin hypersensitivity and recover enamel strength was evaluated using standardized in vitro assessments based on simulated use.

**Methods:**

To assess dentin occlusion a visual occlusion methodology was employed. Dentin discs were subjected to twice-daily simulated brushing (for 5 days) using 0.67 g of toothpaste for 10 s. Simple prototype toothpastes containing SIP-OG and SIP-FF were compared to commercially available controls: Colgate® Sensitive Pro-Relief (CPR) and Sensodyne® Repair and Protect with NovaMin® (SRP). Samples were stored in artificial saliva between treatments. All samples were assessed at baseline and subsequent to each treatment and were scored on a 5-point categorical scale for occlusion. For enamel surface effects, test articles of SIP-OG, SIP-FF, and SIP-FF with NaF were compared to a positive (with NaF) and a negative (no NaF) control paste. Enamel samples were subjected to a pH cycling regime, providing exposure to the toothpaste slurry (i.e., 2 parts deionized water to 1 part toothpaste), mineralizing solution, and demineralizing solution over 5 days of simulated use. Samples were stored overnight in mineralizing solution. Samples were evaluated for fluoride uptake and changes to surface microhardness.

**Results:**

Visual occlusion scores (1 fully occluded to 5 unoccluded) were 2.6, 3.8, 4.4 and 4.0 after 1 day of simulated use for SIP-OG, SIP-FF with NaF, Colgate® Sensitive Pro-Relief and Sensodyne® Repair and Protect, respectively, decreasing to 1.0, 1.8, 3.1 and 3.9 after 5 days of application. SIP-OG provided superior occlusion at the significance level of p ≤ 0.05 at 1, 2, 3, 4, and 5 days. SIP-FF with NaF provided superior occlusion at the significance level of p ≤ 0.05 at 2, 4, and 5 days. Fluoride uptake ranged from 9.0 μg/cm^2^ for SIP-OG to 12.4 μg/cm^2^ for SIP-FF with NaF. Surface microhardness after acid cycling allowed recovery of 59 % of surface microhardness after treatment with SIP-OG or SIP-FF with NaF. SIP-OG achieved significant surface microhardness recovery versus SIP-FF alone, a NaF control paste, and a fluoride free control paste at the significance level of p ≤ 0.05. SIP-FF with NaF achieved surface microhardness recovery versus SIP-FF alone, a NaF control paste, and a fluoride free control paste at the significance level of p ≤ 0.05.

**Conclusions:**

Superior occlusion of dentin tubules was observed with both novel additives compared to commercially available toothpastes. A build-up effect with increasing occlusion was noted with repeated application for both novel additives and ascribed to mineralization effects, as supported by surface microhardness recovery on initial enamel lesions.

## 1 Introduction

Dentin hypersensitivity (DH) is defined as a short sharp pain in response to stimuli including thermal, osmotic, chemical, tactile, or evaporative forces which cannot be attributed to another dental disease [1]. A recent metanalysis by Zeola et. al. *(65 studies)* on the prevalence of DH estimated that 11.5 % of the population suffers from the condition. However, individual studies indicate a prevalence range from 1.3 to 92 % [2]. The effects of DH extend beyond the sensation of pain, as the condition has been correlated with a decrease in oral health-related quality of life measures [3]. The most accepted hypothesis providing a mechanistic basis for the generation of pain in DH is the hydrodynamic theory which succinctly proposes that pain experienced with DH occurs due to the movement of dentinal fluid within exposed dentin tubules [4].

Etiologically, it is believed that DH is the result of the exposure of dentin tissue due to the loss of overlying hard or soft tissues, allowing the painful stimuli to act directly on the tubules [4, 5, 6]. Exposed dentin along the gingival margin is frequently associated with the recession of soft tissues, due to trauma, abrasion from brushing, or periodontal disease and gingivitis. Similarly, dentin may become exposed due to the loss of overlying enamel layer from either mechanical or chemical stresses. In respect of the former, mechanical loss of the enamel layer may be associated with abrasion *(e.g., everyday brushing)*, attrition *(e.g., occlusion wear)*, or abfraction *(e.g., bruxism)* while, in respect of the latter, chemically driven tissue loss is frequently attributable to dietary acid exposures [5]. Along with direct erosion of the enamel layer, acid exposure is known to reduce enamel strength, accelerating the mechanical processes of enamel wear and loss. As these processes are gradual and progressive, the ideal treatment of DH would not only address the symptom of pain at the exposed dentin site, but also provide secondary prevention, that is preventing further areas of dentin exposure following the initial onset of the condition [4, 6].

Arising from its high prevalence, a large variety of at home and clinical use treatments are available for patients suffering with DH [7]. These treatments can be broadly classified in one of two categories. Firstly, there are those agents which aim to treat DH by preventing the action impulse generation in the pain receptors from being produced by fluid flow *(e.g., potassium nitrate (*KNO_3_*))*. The second category related to occlusive agents which serve to occlude dentin tubules, thereby preventing stimuli from causing turbulence in fluid flow [4]. Historically, desensitizing toothpastes containing potassium nitrate (*i.e., KN O*_*3*_) have been the most common treatment offered for DH [8]. Two main criticisms exist for the use of potassium-nitrate based desensitizers. Firstly, clinical data in support of the use of potassium nitrate are inconsistent, with only weak evidence of efficacy [9]. Secondly, KNO_3_ does not provide any secondary prevention against further dentin exposure [6]. In contrast, occlusive agents provide a barrier against dentin fluid flow, either in the form of an overlying film covering the tooth surface, or by directly occluding dentin tubules from within. Accordingly, occlusive agents can provide more rapid pain relief than KNO_3_ based desensitizers. However, for treatment to provide long-term protection against pain, the ideal treatment must occlude exposed dentin tubules *and* provide secondary prevention [4, 6]. A variety of different occlusive agents are considered in the literature for the treatment of DH with various advantages and disadvantages being well documented in established reviews [7].

In respect of occlusive agents, bioactive glasses have emerged as promising candidates for the treatment of DH due to their relative ease of processing, particularly into particles size distributions appropriately matched to occlude tubules directly. In addition, bioactive glasses offer an intrinsic ability to promote biomimetic mineralization reactions [10, 11]. The original bioactive glasses, first developed in the 1970s for the treatment of bony defects, undergo a surface reaction upon exposure to biological fluids, providing the release of constituent ions and the generation of a biosimilar apatite layer [12]. In the dental field, bioactive glasses have been used successfully as bone graft materials to support dentures and implants, as well as in restorative materials such as glass ionomer cements, where they serve both a mechanical reinforcement and as a source of ion release [12]. More recently, bioactive glasses have been used in topical oral health care products both to occlude dentin tubules and support mineralization. For example, NovaMin® *(based on the original 45S5 composition)* has been included in both toothpastes and in office products for the treatment of DH [10]. Subsequently, and as an evolution of this technology, BioMin® F *(which incorporates fluoride into a traditional phosphosilicate glass material)* aims to treat DH through occlusion and is further intended to support mineralization through modulated fluoride release [13, 14]. However, the timeframe of reaction/mineralization of these materials is relatively slow, with studies showing the first evidence of mineralization after 8 and 24 h for BioMin® and NovaMin® glasses respectively [15]. Furthermore, *in vitro* glass research has demonstrated that insoluble glass components are retained during the corrosion process [16], and, as such, the occlusive plug found within the dentin tubules of samples treated with silicate bioactive glasses may retain non-native insoluble silicate materials, and not solely the biomimetic mineral phases they are designed to create. Accordingly, there is a need to further develop the design of bioactive glasses for the treatment of DH. Of particular interest, in this regard, are glasses that are capable of: (i) occluding dentinal tubules, whilst (ii) encouraging rapid mineralization *(in minutes, as opposed to hours and days)* via controlled release of ions *(including fluoride)* and/or synergistic interactions with traditional fluoride sources, (iii) providing low relative dentin abrasion to support daily use and (iv) are completely degradable whilst acting between brushing cycles.

Sensi-IP® is a suite of soluble, next-generation bioactive glasses, intended to address these requirements [17, 18]. From this suite of formulations, two formulations based on borate glass technology have been identified as providing increased degradation relative to traditional silicate bioactive glasses, and, as a result, may provide for more complete dissolution *(between brushing/applications)*, more rapid mineralization, and thus more rapid promotion of enamel repair [17, 18]. The first formulation, Sensi-IP®OG (SIP-OG) is a fluoridated glass, while the second Sensi-IP®FF (SIP-FF) is fluoride-free variant, allowing for additional formulation flexibility *(i.e., for use in combination with monographed fluoride products)* [18]. During this hydrolytic degradation process, glass components, including calcium and fluoride *(optional)*, are released into the local environment, where a pH increase helps promote the precipitation of apatite phases, including fluoridated apatites [19]. Due to the ability of these products to react more quickly, and completely with the aqueous environment of the mouth, they have potential to provide enhanced secondary prevention, by replacing lost tooth mineral content, and shifting the balance towards reprecipitation. As these bioactive glasses promote mineralization during degradation, it can be expected for them to provide not only an initial immediate occlusion but also a build-up effect from repeated application, leading to increased mineralization, and occlusion with repeated uses.

The aim of this paper is to investigate the ability of toothpastes, formulated with the Sensi-IP® formulations *(either SIP-OG and SIP-FF)* to promote enamel repair and provide for the occlusion of dentin tubules. The emphasis of the work is to establish efficacy immediately following application, as well as efficacy following repeated application *(i.e., simulated daily use)*. For visual occlusion testing, simple prototype toothpastes containing SIP-OG and SIP-FF will be compared to commercially available controls: Colgate® Sensitive Pro-Relief (CPR) and Sensodyne® Repair and Protect with NovaMin® (SRP). For enamel mineralization and fluoride uptake, the test articles of SIP-OG, SIP-FF, and SIP-FF with NaF will be compared to a positive (with NaF) and a negative (no NaF) control paste. The first section of this report will investigate the occlusion potential of these novel materials, while the second will investigate the effects on enamel surface properties following simulated use.

## 2 Materials and methods

### 2.1 Preparation of toothpastes

For the purposes of this work, simplified toothpaste formulations containing either (i) SIP-OG *(i.e., fluoridated version)* or (ii) SIP-FF *(i.e., fluoride-free variant)* were prepared according to the formulations presented in Table 1. Where applicable, reagents for the test article toothpastes were sourced as compendial grade (i.e., USP, NF, FCC, or Ph. Eur). Briefly, appropriate quantities of each component were weighed into a 200 ml HDPE vacuum mixing jar and mixed in a Thinky Vacuum Planetary mixer *(Thinky USA, Laguana Hills CA)* at 2000 RPM speed under a 25 kPa vacuum for 18 min. Once mixed, all toothpastes were vacuum sealed until evaluation. Commercial control toothpastes, Sensodyne® Repair and Protect *(lot M9M141)* with NovaMin®, and Colgate® Sensitive Pro Relief *(lot 9737BR1*2CB*)*, as available in the Canadian market, were used as comparators. When applied in slurry form, one part toothpaste was mixed with 2 parts deionized water and mixed for at least 15 min to ensure uniform distribution prior to use.

**Table 1:** Toothpaste formulations (mass %).

Ingredient	5% SIP-FF with NaF Toothpaste	5% SIP-OG toothpaste	5% SIP-FF toothpaste	NaF Control Paste	Negative Control
Glycerol	84.6	84.8	84.8	89.6	89.8
Sodium Laureth Sulfate	1.2	1.2	1.2	1.2	1.2
Silicone Dioxide	7.5	7.5	7.5	7.5	7.5
Sensi-IP FF	5.0	0.0	5.0	0.0	0.0
Sensi-IP OG	0.0	5.0	0.0	0.0	0.0
Carbopol 940	0.5	0.5	0.5	0.5	0.5
Spearmint Oil	1.0	1.0	1.0	1.0	1.0
Sodium Fluoride (NaF)	0.23	0.0	0.0	0.23	0.0

### 2.2 Preparation of artificial saliva

An artificial saliva mixture was prepared as per Takamizawa et al. [20] and refrigerated until use.

### 2.3 Preparation of mineralizing solutions

The remineralizing solution used in this study was prepared as per Huang et al. [21] using 20 mM HEPES, 1.5 mM calcium chloride, 0.9 mM potassium phosphate monobasic, 130 mM potassium chloride, and 1 mM sodium azide. Reagents were dissolved in deionized water at 37 °C, and pH was adjusted to a final value of 7.0 using KOH.

### 2.4 Preparation of demineralizing solution

The demineralizing solution used in this study was prepared as per Huang et al. [21] using 50 mM acetic acid, 2.2 mM calcium nitrate, 2.2 mM potassium phosphate monobasic, and 0.1 ppm sodium fluoride. Reagents were dissolved in deionized water at 37 °C. The pH was adjusted to a final value of 4.5 using NaOH.

### 2.5 Multi time point visual occlusion

#### 2.5.1 Dentin disc preparation for VO

80 slices of dentin (approximately 3 mm square) were prepared from caries-free human molars set in resin discs (polished to p2500 grit) and prepared with dentin tubules perpendicular to the surface. All samples were etched, and baseline checks were performed using scanning electron microscope (Phenom Pro, Thermo Scientific) to confirm patency and sample suitability for the study. Dentin samples were divided into 20 groups of 4, representing the 4 treatment groups (toothpastes) over 5 different timepoints according to Table 2. All dentin discs were soaked in artificial saliva for 60 min prior to the first application. Human samples were obtained and utilized per research ethics procedures at Intertek (Intertek CRS, Cheshire, UK).

**Table 2:** Test conditions for visual dentin occlusion testing.

Treatment Groups	5% SIP-OG toothpaste, 5% SIP-FF with NaF toothpaste, Control Article #1, or Control Article #2				
Number of Treatment Days	1	2	3	4	5
# Treatments/Day	2	2	2	2	2
# Replicate Samples	4	4	4	4	4
Treatment Quantity	0.67 g	0.67 g	0.67 g	0.67 g	0.67 g
Treatment Duration	10 s.	10 s.	10 s.	10 s.	10 s.
Storage between treatments	Artificial saliva	Artificial saliva	Artificial saliva	Artificial saliva	Artificial saliva
Total # Treatments/Time-Point	2	4	6	8	10

#### 2.5.2 Application of toothpaste treatments (VO)

Simulated toothbrushing was performed through a modification of the methods presented by Takamizawa et al [20]. In particular, 0.67 g of the selected toothpaste was applied (100 g load) to 20 dentin discs using an oscillating toothbrush for 10 s (Oral B® Precision). Paste was left to sit for 30 s after brushing, then rinsed thoroughly with deionized water to remove all visible toothpaste remnants. Dentin samples were then returned to soak in artificial saliva at room temperature for at least 1 h, before repeating the application for the second daily application. Following the second daily application, the samples were soaked in artificial saliva for at least 3 h, before storage in dampened tissues overnight. Each day, 4 replicates of each of the toothpastes’ treatment groups (n = 4) were removed from the artificial saliva soaks and allowed to dry at room temperature overnight prior to preparation for SEM.

#### 2.5.3 Imaging

Following overnight air drying, all dentin samples were dried in an oven for 1 h at 37 °C. Samples were sputter-coated with a conductive gold coating and imaged at least 3 times at 3000X magnification using a Phenom ProX SEM. Each 3000X image was scored based on a five-point categorical scale by two single blinded assessors [21, 22, 23]. The grading classification was defined as follows:1) occluded; 2) mostly occluded; 3) equal; 4) mostly unoccluded; and 5) unoccluded.

### 2.6 Enamel fluoride uptake

#### 2.6.1 Sample preparation

Enamel blocks *(ca.* 4 mm *×* 4 mm*)* were prepared from the labial surface of bovine incisors. Enamel was lapped and polished to a fineness of 0.3 μm using aluminum oxide slurries to achieve a smooth flat surface. Samples were then coated with nail varnish, to leave a 3 mm × 3 mm window of exposed enamel and mounted in a crystalizing dish using double-sided tape. Enamel samples were covered in a 2.5 cm thick layer of methylcellulose gel and set overnight before being covered in a 2.5 cm thick layer of 0.1 M lactic acid and incubated at 37 °C for 4 days. Following acid-etching, samples were thoroughly rinsed to remove acid and gel layer.

#### 2.6.2 Fluoride retrieval

Enamel fluoride content was measured on the etched samples to establish their baseline fluoride levels then again after pH cycling treatment. Briefly, enamel samples were coated with 50 μL of 1 M perchloric acid for 10 min. The acid was retrieved using vacuum suction, followed by 3 rinses with 50 μL of 1 M sodium acetate solution, which were added to the retrieved acid sample. The retrieved acid solution was diluted 1 to 1 with TISAB II and fluoride content measured using an Orion fluoride ion-selective electrode, calibrated with standard solutions of 1, 10, 20 and 100 ppm (m/m) F^−^.

#### 2.6.3 pH cycling

10 enamel samples were assigned to each of the toothpaste's treatment group (n = 10) and subjected to a pH cycling regime adapted from Karlinsey et al., including alternating immersion on toothpaste slurries, remineralizing solution, and demineralizing solution as per Table 3 [24].

#### 2.6.4 Calculation of fluoride uptake

The fluoride uptake of the samples following the pH cycling treatment was calculated relative to the exposed surface area of enamel as follows:

$$\begin{array}{l} {\left[Fluoride\right]}_{lesion}\left(mg/L\right)={\left[Fluoride\right]}_{post-treatment}\left(mg/L\right)-{\left[Fluoride\right]}_{baseline}\left(mg/L\right) \end{array}\;\;\begin{array}{l} Fluoride_{total}\left(\mu g\right)={\left[Fluoride\right]}_{lesion}\left(mg/L\right)\times solutionvolume\left(mL\right)\\ \begin{array}{l} FluorideUptake\left(\frac{\mu g}{cm^{2}}\right)=\frac{Fluoride_{total}\left(\mu g\right)}{Exposedareaofenamel\left(cm^{2}\right)} \end{array} \end{array}\;\;$$

### 2.7 Enamel surface microhardness assessment

#### 2.7.1 Sample preparation

Enamel blocks shaped to approximately 4 by 4 mm were sliced from the labial surface of bovine incisors, lapped and polished to a grit of 0.04 μm. One corner was abraded off to allow for sample orientation, and samples were stored, refrigerated, and dampened with 0.1% thymol solution until use. Baseline surface microhardness (SMH) measurements were assessed as per 2.4.2, with an inclusion criterion of a SMH of ≥250 Knoop Hardness (HK), and standard deviation of ≤20. Following baseline assessment, an initial demineralization challenge was applied by soaking the samples in 8 ml of demineralization solution per block at 37 °C for 60 min, followed by a deionized water rinse.

#### 2.7.2 Surface microhardness measurements

Surface microhardness measurements were taken for each enamel block both before demineralization as a quality check for inclusion in the study, after initial demineralization treatment, and following pH cycling treatment (Table 3). 10 enamel block samples were assigned to each of the toothpaste's treatment group (n = 10). Surface microhardness was analyzed using a series of 8 indents made at 100 μm spacing using a 50 g load and 10 s dwell time. Measurements of the indents were taken using a 50 X objective, and hardness was expressed as Hardness Knoop.

**Table 3:** pH cycling regime for enamel fluoride uptake and surface microhardness testing. A deionized water rinse was applied between each step.

Step	Solution	Volume	Duration
1	Toothpaste slurry	5 ml	2 min
2	Remineralizing solution	20 ml	58 min
3	Toothpaste slurry	5 ml	2 min
4	Remineralizing solution	20 ml	58 min
5	Demineralizing solution	20 ml	60 min
6	Remineralizing solution	20 ml	120 min
7	Toothpaste Slurry	5 ml	2 min
8	Remineralizing solution	20 ml	58 min
9	Toothpaste Slurry	5 ml	2 min
10	Remineralizing solution	20 ml	Overnight
11	Repeat Steps 1–10 4 more times (5 days total)		

#### 2.7.3 Calculation of SMH recovery

$$\%\;SMHR=100\times \;\left[\frac{SMHfinal-SMH\;Demineralized}{SMH\;Baseline-SMH\;Demineralized}\right]$$

### 2.8 Statistics

All statistical analysis was performed using Minitab 18 software. For each experiment, summary statistics were generated for each treatment group and timepoint *(e.g., n, mean, standard deviation)*. All data sets were tested for normality using the Anderson-Darling test. Pairwise comparison was performed between treatment groups for each experiment and timepoint. For the enamel surface microhardness experiments, all data sets satisfied the assumptions criteria, and one-way ANOVA was used to compare experimental results. For the visual occlusion experiment and fluoride-uptake tests, 2-sample t-tests were used to make pairwise comparisons between occlusion scores when assumptions of normality could be met, and a Mann-Whitney test was used to make comparisons when one or more of the pairs failed the normality test. All statistical tests were performed at a 0.05 significance level (i.e., p ≤ 0.05).

## 3 Results

### 3.1 Multi timepoint visual occlusion

**Figure 1:**
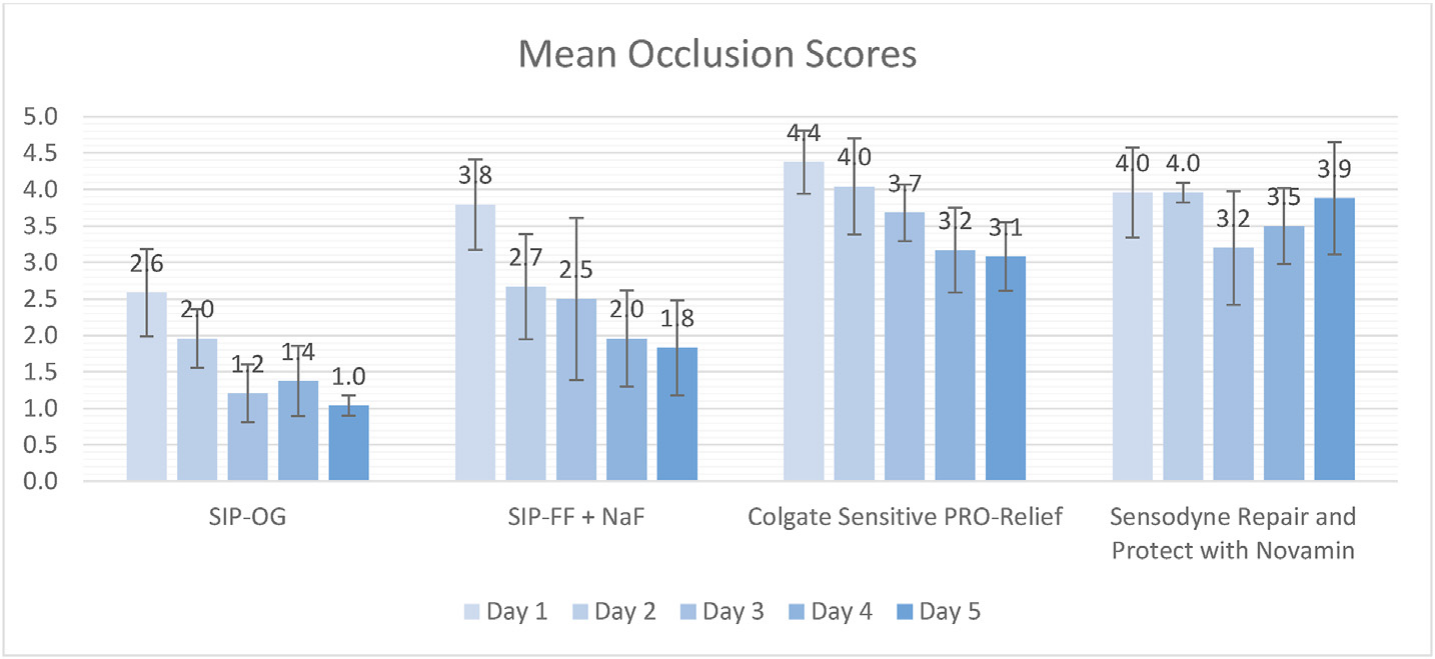
Multi day visual occlusion scores over 5 days of simulated twice daily brushing (mean values ± standard deviations).

Visual occlusion scores after a single day of simulated brushing ranged from 4.4 for CPR to 2.6 for the SIP-OG toothpaste formulation (Figure 1). The SIP-OG toothpaste formulation was the best-performing toothpaste at all timepoints investigated, providing significantly superior occlusion to both commercial toothpastes. The SIP-FF with NaF formulation toothpaste was the next best-performing toothpaste, demonstrating significantly greater occlusion after 2, 4 and 5 days of occlusion when compared to both commercial toothpastes *(Colgate*® *Sensitive- Pro-Relief (CPR) and Sensodyne*® *Repair and Protect with NovaMin*® *(SRP))*. A trend towards increasing visual occlusion with repeated application was observed for both SIP-OG and SIP-FF with NaF toothpastes as well as the CPR toothpaste, but not for the SRP formulation. Following 5 days of simulated use, visual occlusion scores ranged from 3.9 for SRP to 1.0 for SIP-OG.

### 3.2 Enamel fluoride uptake

Fluoride uptake measurements (Table 4) of 9.0 ± 3.4 μg/cm^2^ were observed for samples treated with the toothpaste containing 5% SIP-OG *(ca.* 800 ppm *fluoride)*. Fluoride uptake measurements of 12.4 ± 4.9 μg/cm^2^ were observed following treatment with the toothpaste containing SIP-FF with NaF (1040 ppm fluoride) and measurements of 24.2 ± 4.9 μg/cm^2^ were reported following treatment with the NaF control paste (1040 ppm F^−^). No significant difference was found between the two SIP-containing pastes.

**Table 4:** Enamel fluoride uptake results following 5 days of pH cycling and treatment in toothpaste slurries (∗ means which share a letter are not significantly different).

Toothpaste	Fluoride Content	Mean Fluoride Uptake (μg/cm^2^)	Statistical Comparison Groupings∗	Mean Percentage of surface microhardness recovery (SMHR)	Statistical Comparison Groupings∗
5% SIP-OG	800 ppm	9.0 ± 3.4	B	58.8 ± 16.1%	A
5% SIP-FF + NaF	1040 ppm	12.4 ± 4.9	B	58.9 ± 14.6%	A
5% SIP-FF	0 ppm	N/A	N/A	5.8 ± 18.8%	B, C
Positive Control (NaF Control)	1040 ppm	24.2 ± 4.9	A	23.3 ± 24.9%	B
Negative Control (Blank Control)	0 ppm	0.4 ± 0.1	C	-6.3 ± 14.2%	C

### 3.3 Enamel surface microhardness recovery

Enamel surface microhardness recovery (Table 4) was observed to be 5.8% ± 18.8% when treated with 5% SIP-FF alone (0 ppm F^*-*^) and increased to 58.9 ± 14.6% when treated with a toothpaste formulated with SIP-FF and NaF (1040 ppm F^*-*^). Similar results of 58.8 ± 16.1% surface microhardness recovery was observed following treatment with 5% SIP-OG toothpaste (800 ppm F^*-*^). Positive *(*1040 ppm F^*-*^*)* and negative *(*0 ppm F^*-*^*)* control pastes resulted in surface microhardness recoveries of 23.3 ± 24.9% and -6.3 ± 14.2%. No significant difference was found between the SIP-OG and SIP-FF with NaF toothpastes, which were both significantly greater than the SIP-FF, the NaF *(positive control)* pastes and negative control pastes.

## 4 Discussion

**Figure 2:**
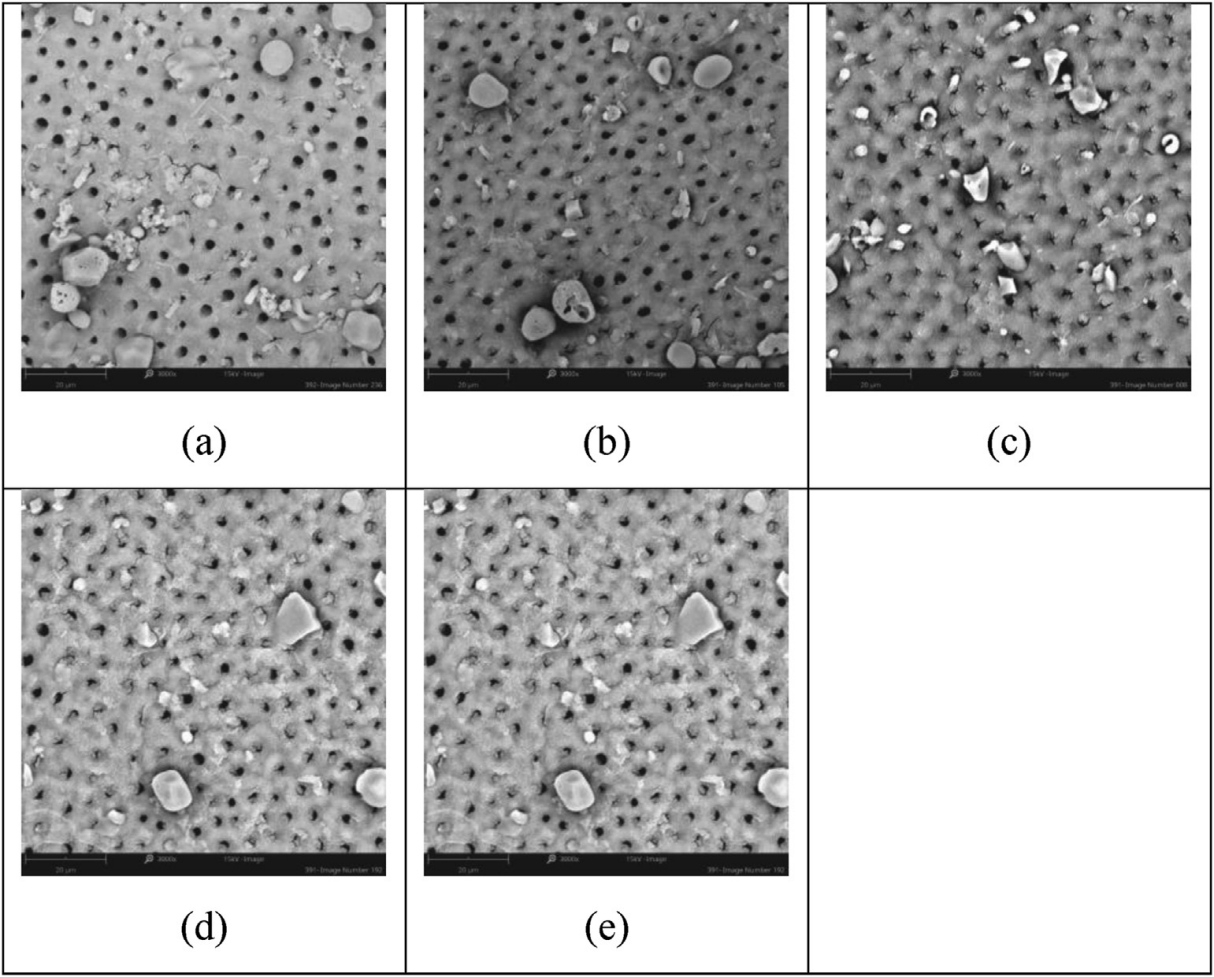
Representative SEM images of dentin samples treated with 5% SIP-FF + NaF paste after: a) 1 day simulated use, mean assessor score 3.8; b) 2 days simulated use, mean assessor score 2.7; c) 3 days simulated use, mean assessor score 2.5; d) 4 days simulated use mean assessor score 2.0; and e) 5 days simulated use, mean assessor score 1.8.

**Figure 3:**
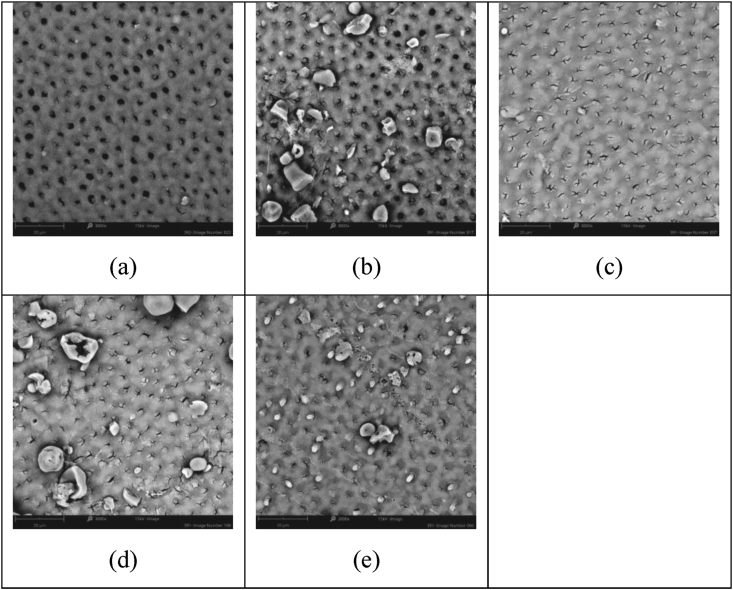
Representative SEM images of dentin samples treated with 5% SIP-OG paste after: a) 1 day simulated use, mean assessor score 2.6; b) 2 days simulated use, mean assessor score 2.0; c) 3 days simulated use, mean assessor score 1.2; d) 4 days simulated use mean assessor score 1.4; and e) 5 days simulated use, mean assessor score 1.0.

**Figure 4:**
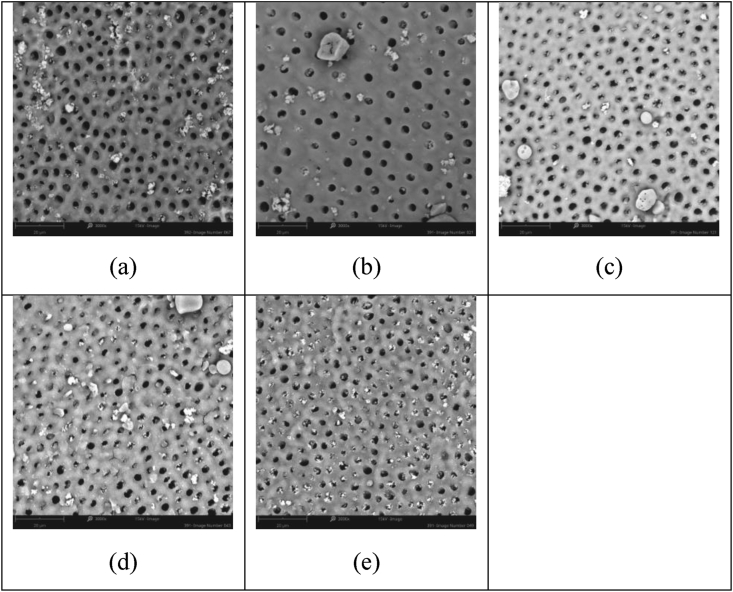
Representative SEM images of dentin samples treated with CPR after: a) 1 day simulated use, mean assessor score 4.4; b) 2 days simulated use, mean assessor score 4.0; c) 3 days simulated use, mean assessor score 3.7; d) 4 days simulated use mean assessor score 3.2; and e) 5 days simulated use, mean assessor score 3.1.

**Figure 5:**
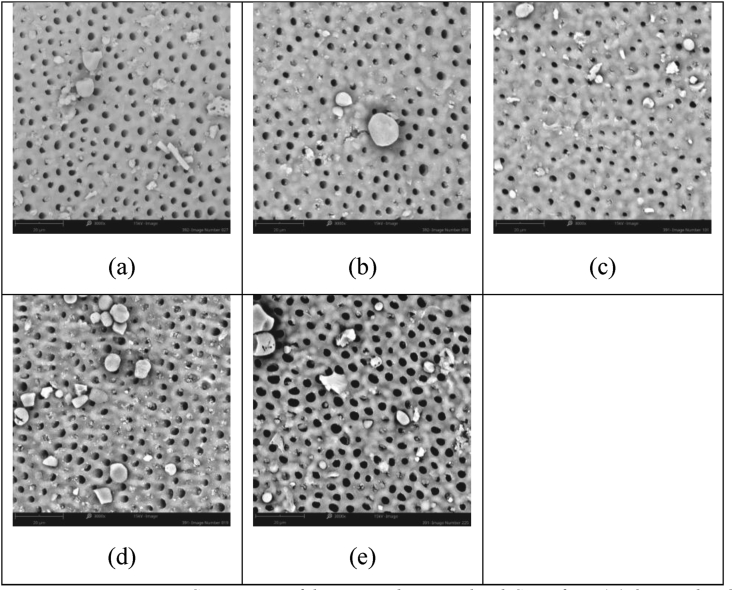
Representative SEM images of dentin samples treated with SRP after: a) 1 day simulated use, mean assessor score 4.0; b) 2 days simulated use, mean assessor score 4.0; c) 3 days simulated use, mean assessor score 3.2; d) 4 days simulated use mean assessor score 3.5; and e) 5 days simulated use, mean assessor score 3.9.

This investigation focused on the study of two novel additives; firstly, fluoridated SIP-OG glass which is designed to allow for 800 ppm F^−^ when formulated at a 5 wt% loading into a toothpaste and, secondly, non-fluoridated SIP-FF glass, has been designed to allow for flexibility of formulation to achieve the intended use and meet associated regulatory requirements. The non-fluoridated version, in particular, allows for the tailoring of toothpaste formulations to various total fluoride contents, with different fluoride compounds and concentrations *(e.g., NaF or SnF*_*2*_*)*. In contrast, the low fixed fluoride concentration of the SIP-OG glass has been formulated to provide effectiveness in occlusion while supporting enamel repair and increasing the margin of safety through minimized fluoride loads. Both SIP-OG and SIP-FF containing toothpastes demonstrated high levels of visual occlusion after single and repeated applications in this study and were superior to commercially available pastes. Notably, after 5 days of twice-daily treatment with SIP-OG containing paste a visual occlusion score of 1.0, representing complete occlusion of the tubules, was observed by blinded assessors. The commercial control pastes unexpectedly scored between 4 to 3 on the visual occlusion scale, leaving dentin tubules mostly unoccluded to partially occluded. Based on hydrodynamic theory, the prototype toothpaste chassis developed with each variant of Sensi-IP® would provide a more significant and more rapid reduction in fluid flow, and hence pain perception, compared to commercial controls. It is of note however, that the 5 days of simulated use is less than the 8-week clinical trial period most often used to evaluate the efficacy of marketed pastes [25]. Therefore, the short duration of the test may not have provided sufficient time or repeated applications for commercial controls to achieve equivalence to the Sensi-IP® containing toothpastes. Reductions in visual occlusion scores with repeated applications were greatest for the SIP-OG and SIP-FF with NaF toothpastes. This buildup effect observed with both SIP-OG and SIP-FF with NaF was the result of small intratubular deposits, and not the accumulation of bioactive SIP-OG or SIP-FF on the dentin surfaces (Figure 2 and Figure 3). This accumulation of intratubular deposits is attributable to the more rapid reaction of the Sensi-IP® glass formulations relative to traditional bioactive glasses such as NovaMin®, allowing for more rapid generation of mineral deposits within the dentin tubules. Contrastingly, only modest reductions were observed with repeated applications of the Colgate® product and no reduction seen with repeated application of SRP which may have adverse implications for patient adherence (Figure 4 and Figure 5) [26,27].

With respect to the visual occlusion data, direct comparable data are not available in the literature due to variability in brushing techniques across the literature. Notably, this study utilized a 10-second application and rinsing steps, while most literature studies utilize 2 min of brushing on a single surface and may or may not rinse the dentin surface. The 10-second interval was utilized to simulate actual use and is more clinically representative whilst being suitable to investigate immediate effects. While 2 min of brushing is generally recommended as part of an oral health routine, this time is allocated to all tooth surfaces, with the brushing of a single location expected to be significantly shorter. For example, Ganns et al. revealed an average brushing duration of only 96 s, which, when divided into 12 surface regions of the lingual, buccal and occlusal surfaces of the 4 quadrants, would result in an average 8 s per surface region [28]. Despite the lack of direct comparable data, an in vitro investigation, comparing a NovaMin® containing toothpaste to one containing BioMin® F (a fluoridated phosphosilicate glass), reported 79 and 88% occlusion respectively for the pastes when no rinsing step was used following a 2-minute brushing application, with a minor increase to 81 and 89% occlusion following immersion in an artificial saliva [14]. Similar occlusion scores were reported by Bakri et al. for both NovaMin® and arginine toothpastes after 2 min of brushing with 5-point occlusion scores of 1.5 and 2.25 respectively [21]. Longer brushing durations do appear to improve occlusion scores. However, caution must be used when interpreting such studies, as excessive brushing force or duration (i.e., 2 min on a single tooth surface) are known to be etiological factors for the progression of DH and should, therefore, be strongly discouraged [6]. An *in situ* study conducted comparing the CPR toothpaste to the strontium acetate paste similarly used a 10-second brushing application (performed *ex vivo*) on a buccal appliance which was worn between brushing for 4 h, as well as for 60 min after application, to allow for reaction with the oral environment. In that study, an occlusion score of approximately 2.5 was achieved for the arginine toothpaste after one day of twice-daily applications [29]. While experimental results from this study show lesser occlusion levels than previously reported for SRP as well as CPR formulas, they remain consistent in the directional superiority of the Sensodyne® product at early timepoints, agreeing with the overall trend of the published data supporting the conclusion of superior occlusion with the Sensi-IP® formulation than the commercially available toothpastes.

Enamel fluoride uptake was greatest with the control toothpaste containing 1040 ppm fluoride as NaF. However, this did not translate into significant improvements in surface microhardness. While a reduction in fluoride availability was seen in the toothpaste which incorporated 5% SIP-FF with 1040 ppm F^−^ *(as NaF)*, the inclusion of SIP-FF did result in a significant increase in surface microhardness. This effect appears to be from a synergistic effect of SIP-FF and NaF, as no significant increase in surface microhardness was observed for treatment with SIP-FF alone. The reduction in fluoride uptake of the two Sensi-IP® containing toothpastes relative to the 1040 ppm fluoride toothpaste did not, however, adversely effect surface microhardness, and may in part be attributable to calcium and fluoride interactions as well as the reduced concentration of F^−^ in the SIP-OG formulated pastes. In respect of the former, toothpaste formulations used in the evaluation of enamel surface effects were not optimized for fluoride release or mineralization; rather they were selected as simple chassis to allow for the isolation of the effects of the Sensi-IP® variants. Interactions between calcium compounds and fluoridated toothpastes have been recognised to reduce fluoride availability, likely due to the production of insoluble CaF_2_ before enamel uptake can occur. Stabilized toothpastes containing calcium within their formulations *(most commonly calcium carbonate abrasives)* have been formulated with sodium monoflurophosphate which prevents the undesirable interaction between the calcium and fluoride. Such a formulation is used for the production of SRP which contains a calcium-containing bioactive glass, and 1450 ppm F^−^ as sodium monofluorophosphate as the fluoride source. The formulation of a SIP-FF toothpaste formulated with sodium monofluorophosphate as the fluoride source should be investigated to better understand the interaction between fluoride source, surface microhardness and fluoride uptake in conjunction with SIP-FF use.

A notable finding was that fluoride uptake and surface microhardness results were statistically equivalent for both the SIP-FF with 1040 ppm fluoride toothpaste and the 5% SIP-OG toothpaste formulation which contains 800 ppm fluoride from the bioactive glass despite the differences in fluoride loading. A notable difference between these formulations would be the gradual release of fluoride from the SIP-OG glass relative to the quick burst release from NaF in solution [17, 19]. Slower release of F^−^ over time may provide for equivalent fluoride availability and mineralization support, as seen in this study, while minimizing toxicity concerns associated with the element.

Finally, and in respect of limitations of the study, the authors note that standardized test variables are limited in the literature, and while every effort has been made to replicate the real-world use of a toothpaste in this work, the simulated nature of this type of research presents intrinsic limitations. On the other hand, tubule occlusion is regarded as a principal methodology to assess the likely clinical success of new desensitizing agent. As such, and based on the superior performance observed in this study, the authors are now preparing to complete a clinical study for these test articles.

## 5 Conclusion

Simplified toothpastes formulated with two variants of Sensi-IP® were examined in this study. These test toothpastes provided superior dentin occlusion versus commercial products at a single day of twice daily treatment and with continued use over 5 days of simulated brushing. The commercial articles resulted in mostly unoccluded to partially occluded, consistent with published literature. The test toothpastes also showed superior recovery in enamel microhardness when treated with Sensi-IP® versus NaF alone. The rapid onset of occlusion coupled with rapid promotion of enamel repair could be beneficial for the treatment of DH to promote patient satisfaction and compliance.

## Declarations

### Author contribution statement

Kathleen MacDonald: Conceived and designed the experiments; Wrote the paper.

Effie Boudreau: Contributed reagents, materials, analysis tools or data; Wrote the paper.

Gavin Vaughan Thomas, Thomas Charles Badrock, Luke John Davies, Michael James Lloyd, Paul Steven Spradbery: Performed the experiments; Analyzed and interpreted the data.

Stephanie Turner-Cahill: Analyzed and interpreted the data; Wrote the paper.

Daniel Boyd: Conceived and designed the experiments; Wrote the paper.

### Funding statement

This research did not receive any specific grant from funding agencies in the public, commercial, or not-for-profit sectors.

### Data availability statement

Data included in article/supplementary material/referenced in article.

### Declaration of interests statement

The authors declare the following conflict of interests: Daniel Boyd, Effie Boudreau, Kathleen MacDonald are inventors of Sensi-IP®. Daniel Boyd, Effie Boudreau, Kathleen MacDonald and Stephanie Turner-Cahill are shareholders and/or option holders in IR-Scientific.

### Additional information

No additional information is available for this paper.
